# Supplementary material for: PNPLA3 and TM6SF2 exacerbate the impact of alcohol and metabolic dysfunction on liver fibrosis
Source: JHEP Rep. 2025 Oct 30;8(1):101649. doi: 10.1016/j.jhepr.2025.101649 (PMC12765432; doi:10.1016/j.jhepr.2025.101649)
Supplement: Multimedia component 1 [file mmc1.pdf]

PNPLA3 and TM6SF2 exacerbate the impact of alcohol and metabolic dysfunction on liver fibrosis

Sophie Gensluckner, Helle Lindholm Schnefeld, Jan Embacher, Camilla Dalby Hansen, Lorenz Balcar, Katrine Tholstrup Bech, Paul Thöne, Nikolaj Torp, Bernhard Wernly, Laura Maarit Pikkupeura, Stephan Zandanell, Christian Datz, Michael Strasser, Mads Israelsen, Mattias Mandorfer, Torben Hansen, Aleksander Krag, Elmar Aigner, Maja Thiele, Georg Semmler

Table of contents

Fig. S1……………………………………………………………………………......2

Table S1…………………………………………………………………………………3

Table S2…………………………………………………………………………………5

Table S3…………………………………………………………………………………6

Supplementary references…………………………………………………………....7

Fig. S1. Patient flowchart for the tertiary-care-cohort (n=1554) and at-risk-cohort (n=1728).

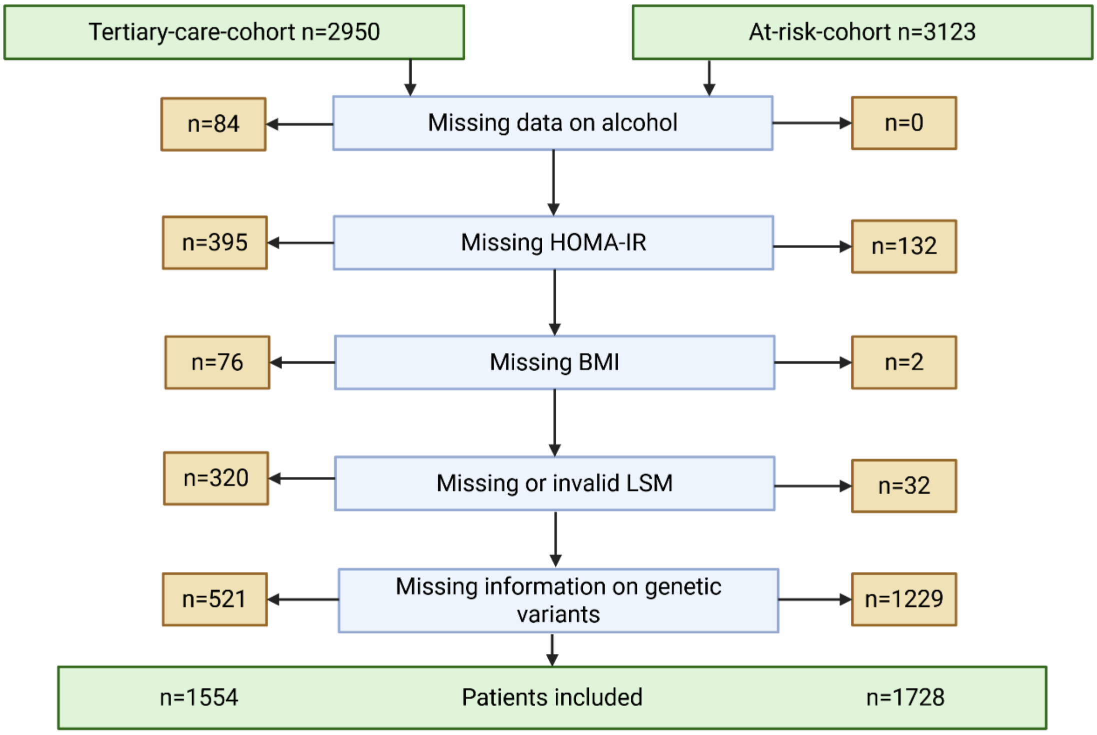

Abbreviations: HOMA-IR – Homeostatic model assessment or insulin resistance; BMI – body mass index; LSM – liver stiffness measurement

Table S1. Distribution of genetic risk variants in European populations the literature and the current study cohorts. Cohorts were sorted in respective settings according to minor allele frequency (MAF).

**Gene**	**Setting**	**Study**	**Wildtype (%)**	**Heterozygous minor allele (%)**	**Homozygous minor allele (%)**	**MAF (%)**
PNPLA3 rs738409	General population	[1]	66	31	2.8	0.18
		[2, 3] (UK Biobank)	62	34	4.7-4.8	0.22
		1000 Genomes Project[1]	-	-	-	0.23
		[4]	-	-	-	0.23
		[5]	59	38	3.8	0.23
		[3]	60	35	5.1	0.23
		[6]	60	36	4.6	0.23
		[7]	-	-	-	0.24
		[8]	53	39	8.4	0.28
	At-risk population	**At-risk cohort**	**56**	**37**	**6.6**	**0.25**
		[9]	57	34	9.4	0.26
	Tertiary care	**Tertiary-care-cohort**	**52**	**39**	**9.5**	**0.29**
		[1]	41-44	44-45	12-14	0.34-0.37
		[10]	44	43	14	0.36
		[11]	42	43	15	0.37
		[12] (median + range from meta-analysis)	38 (25-78)	41 (15-49)	17 (6.9-36)	0.40
TM6SF2 rs58542926	General population	[13]	-	-	-	0.07
		1000 Genomes Project[1]	-	-	-	0.07
		[7]	-	-	-	0.07
		[6]	81	14	0.7	0.08
		[8]	84	15	0.6	0.08
		[3] (UK Biobank)	85	14	0.6	0.08
		[3]	83	16	0.8	0.09
	At-risk population	**At-risk cohort**	**85**	**14**	**0.6**	**0.08**
	Tertiary care	**Tertiary-care-cohort**	**82**	**17**	**0.8**	**0.09**
		[11]	79	19	1.8	0.11
		[10]	78	20	2.3	0.12
MBOAT7	General population	[14][4]	34	49	18	0.42
		[7]	-	-	-	0.43
		1000 Genomes Project[1]	-	-	-	0.44

	At-risk population	**At-risk cohort**	**33**	**49**	**18**	**0.42**
		[15]	30	52	17	0.43
	Tertiary care	**Tertiary-care-cohort**	**33**	**46**	**21**	**0.44**
		[14]	33	46	21	0.44
		[16]	34	45	21	0.44
		[17]	29	50	21	0.46
		[11]	31	47	22	0.46
HSD17B13 rs72613567 (rs10433937)[3]	General population	[3] (UK Biobank)	48	43	9.6	0.31
		[3]	50	40	7.8	0.28
	At-risk population	**At-risk cohort**	**50**	**41**	**8.3**	**0.29**
	Tertiary care	**Tertiary-care-cohort**	**58**	**36**	**6.8**	**0.25**
		[18]	-	-	-	0.21
		[19]	-	-	-	0.19
SERPINA1 rs28929474	General population	1000 Genomes Project[1]	-	-	-	0.02
		[20] (UK Biobank)	96	4	0.04	0.02
	At-risk population	**At-risk cohort**	**96**	**4.2**	**0**	**0.02**
	Tertiary care	[21]	96	3.8	0.14	0.02
		**Tertiary-care-cohort**	**94**	**6.3**	**-[5]**	**0.03[5]**

[1] Derived from European population (n=503) from the 1000 Genomes project, assessed from https://ldlink.nih.gov/; [2] High linkage disequilibrium, $r^2=0.99$; [3] High linkage disequilibrium, $r^2=0.99$; [4] Based on data from European Americans; [5] As carrying the homozygous risk allele denotes alpha-1 antitrypsin deficiency, these individuals were a priori excluded from the study cohort. MAF was calculated assuming n=0 for Z/Z.

Abbreviations: *PNPLA3* - Patatin-like phospholipase domain-containing protein 3, *TM6SF2* - Transmembrane 6 superfamily 2, *MBOAT7* - Membrane Bound O-Acyltransferase Domain Containing 7, *HSD17B13* - Hydroxysteroid 17-Beta Dehydrogenase 13, *SERPINA1* - Serpin Family A Member 1, MAF - minor allele frequency

Table S2. Univariable linear regression analysis studying the association of genetic risk variants with liver fibrosis assessed by LSM (log-transformed).

	Tertiary-care-cohort (n=1554)			At-risk-cohort (n=1728)		
	**Estimate (β)**	**Standard Error**	**p-value**	**Estimate (β)**	**Standard Error**	**p-value**
PNPLA3 G-allele	0.145	0.029	**5.24×10^{-7}**	0.061	0.020	**0.002**
TM6SF2 T-allele	0.113	0.038	**0.003**	0.054	0.028	0.054
HSD17B13 TA-allele (A-allele)	0.020	0.029	0.495	0.004	0.020	0.848
MBOAT7 T-allele (C-allele)	0.038	0.039	0.323	0.012	0.021	0.561
SERPINA1 Z-allele	-0.120	0.061	**0.047**	0.050	0.050	0.315

Abbreviations: LSM – liver stiffness measurement, *PNPLA3* - Patatin-like phospholipase domain-containing protein 3, *TM6SF2* - Transmembrane 6 superfamily 2, *MBOAT7* - Membrane Bound O-Acyltransferase Domain Containing 7, *HSD17B13* - Hydroxysteroid 17-Beta Dehydrogenase 13, *SERPINA1* - Serpin Family A Member 1, MAF - minor allele frequency

Table S3. Multivariable linear regression investigating factors associated with liver fibrosis assessed by LSM (log-transformed) with and without considering an interaction / effect modification of genetic risk variants *PNPLA3* and *TM6SF2* and alcohol (semi-quantitatively), insulin resistance (as assessed by HOMA-IR ≥2.5) and obesity (BMI ≥30kg/m²).

	**Tertiary-care-cohort (n=1554)**			**At-risk-cohort (n=1728)**		
	**Estimate (β)**	**Standard Error**	**p-value**	**Estimate (β)**	**Standard Error**	**p-value**
**Multivariable regression with interaction term**						
Age, per 10 years	0.076	0.008	**$< 2\times10^{-16}$**	0.019	0.009	**0.033**
Female sex	-0.053	0.026	**0.039**	-0.138	0.019	**4.96×10^{-13}**
Obesity (BMI ≥30kg/m²)	0.231	0.047	**1.01×10^{-6}**	0.138	0.029	**2.98×10^{-6}**
Insulin resistance (HOMA-IR ≥2.5)	0.193	0.039	**9.33×10^{-7}**	0.100	0.029	**0.001**
Alcohol, 20-50/30-60g/d	0.125	0.053	**0.019**	-0.04	0.033	0.218
Alcohol, >50/60g/d	0.282	0.061	**4.60×10^{-6}**	0.062	0.046	0.180
TM6SF2 T-allele	0.032	0.047	0.496	-0.043	0.052	0.407
TM6SF2 × Obesity	0.117	0.077	0.126	0.07	0.058	0.228
TM6SF2 × Insulin resistance	-0.069	0.068	0.315	0.093	0.058	0.108
TM6SF2 × Alcohol, 20-50/30-60g/d	-0.033	0.097	0.738	0.050	0.067	0.457
TM6SF2 × Alcohol, >50/60g/d	0.447	0.105	**2.01×10^{-5}**	-0.037	0.098	0.708
PNPLA3 G-allele	0.02	0.036	0.576	-0.027	0.039	0.48
PNPLA3 × Obesity	-0.157	0.062	**0.011**	0.021	0.042	0.624
PNPLA3 × Insulin resistance	0.227	0.053	**1.75×10^{-5}**	0.082	0.042	**0.05**
PNPLA3 × Alcohol, 20-50/30-60g/d	0.019	0.070	0.783	0.029	0.047	0.536
PNPLA3 × Alcohol, >50/60g/d	0.316	0.081	**0.0001**	0.123	0.064	0.055
	**Adjusted R^2: 0.288**			**Adjusted R^2: 0.132**		

Abbreviations: LSM – liver stiffness measurement, *PNPLA3* - Patatin-like phospholipase domain-containing protein 3, *TM6SF2* - Transmembrane 6 superfamily 2, HOMA-IR - Homeostatic model assessment or insulin resistance, BMI – body mass index

Supplementary references

Author names in bold designate shared co-first authorship

[1] Valenti L, Al-Serri A, Daly AK, et al. Homozygosity for the patatin-like phospholipase-3/adiponutrin I148M polymorphism influences liver fibrosis in patients with nonalcoholic fatty liver disease†. Hepatology (Baltimore, Md) 2010;51:1209-1217.

[2] Kim H-s, Xiao X, Byun J, et al. Synergistic Associations of PNPLA3 I148M Variant, Alcohol Intake, and Obesity With Risk of Cirrhosis, Hepatocellular Carcinoma, and Mortality. JAMA network open 2022;5:e2234221-e2234221.

[3] Gellert-Kristensen H, Richardson TG, Davey Smith G, et al. Combined Effect of PNPLA3, TM6SF2, and HSD17B13 Variants on Risk of Cirrhosis and Hepatocellular Carcinoma in the General Population. Hepatology (Baltimore, Md) 2020;72:845-856.

[4] Kozlitina J, Sookoian S. Global Epidemiological Impact of PNPLA3 I148M on Liver Disease. Liver international : official journal of the International Association for the Study of the Liver 2025;45:e16123.

[5] Romeo S, Kozlitina J, Xing C, et al. Genetic variation in PNPLA3 confers susceptibility to nonalcoholic fatty liver disease. Nature genetics 2008;40:1461-1465.

[6] Köpp J, Fleßa S, Lieb W, et al. Association of PNPLA3 rs738409 and TM6SF2 rs58542926 with health services utilization in a population-based study. BMC Health Services Research 2016;16:41.

[7] Simons N, Isaacs A, Koek GH, et al. PNPLA3, TM6SF2, and MBOAT7 Genotypes and Coronary Artery Disease. Gastroenterology 2017;152:912-913.

[8] **Semmler G, Balcar L**, Wernly S, et al. No association of NAFLD-related polymorphisms in PNPLA3 and TM6SF2 with all-cause and cardiovascular mortality in an Austrian population study. Wiener klinische Wochenschrift 2024;136:251-257.

[9] Kantartzis K, Peter A, Machicao F, et al. Dissociation between fatty liver and insulin resistance in humans carrying a variant of the patatin-like phospholipase 3 gene. Diabetes 2009;58:2616-2623.

[10] Liu YL, Reeves HL, Burt AD, et al. TM6SF2 rs58542926 influences hepatic fibrosis progression in patients with non-alcoholic fatty liver disease. Nat Commun 2014;5:4309.

[11] Krawczyk M, Rau M, Schattenberg JM, et al. Combined effects of the PNPLA3 rs738409, TM6SF2 rs58542926, and MBOAT7 rs641738 variants on NAFLD severity: a multicenter biopsy-based study1. Journal of lipid research 2017;58:247-255.

[12] Zhao Y, Zhao W, Ma J, et al. Patatin-like phospholipase domain-containing 3 gene (PNPLA3) polymorphic (rs738409) single nucleotide polymorphisms and susceptibility to nonalcoholic fatty liver disease: A meta-analysis of twenty studies. Medicine 2023;102:e33110.

[13] Kozlitina J, Smagris E, Stender S, et al. Exome-wide association study identifies a TM6SF2 variant that confers susceptibility to nonalcoholic fatty liver disease. Nat Genet 2014;46:352-356.

[14] **Mancina RM, Dongiovanni P**, Petta S, et al. The MBOAT7-TMC4 Variant rs641738 Increases Risk of Nonalcoholic Fatty Liver Disease in Individuals of European Descent. Gastroenterology 2016;150:1219-1230.e1216.

[15] Luukkonen PK, Zhou Y, Hyötyläinen T, et al. The MBOAT7 variant rs641738 alters hepatic phosphatidylinositols and increases severity of non-alcoholic fatty liver disease in humans. Journal of hepatology 2016;65:1263-1265.

[16] Donati B, Dongiovanni P, Romeo S, et al. MBOAT7 rs641738 variant and hepatocellular carcinoma in non-cirrhotic individuals. Scientific Reports 2017;7:4492.

[17] Thangapandi VR, Knittelfelder O, Brosch M, et al. Loss of hepatic Mboat7 leads to liver fibrosis. Gut 2021;70:940-950.

[18] Anstee QM, Darlay R, Cockell S, et al. Genome-wide association study of non-alcoholic fatty liver and steatohepatitis in a histologically characterised cohort(☆). Journal of hepatology 2020;73:505-515.

[19] Ma Y, Belyaeva OV, Brown PM, et al. 17-Beta Hydroxysteroid Dehydrogenase 13 Is a Hepatic Retinol Dehydrogenase Associated With Histological Features of Nonalcoholic Fatty Liver Disease. Hepatology (Baltimore, Md) 2019;69:1504-1519.

[20] Hakim A, Moll M, Qiao D, et al. Heterozygosity of the Alpha 1-Antitrypsin Pi*Z Allele and Risk of Liver Disease. Hepatology communications 2021;5:1348-1361.

[21] Strnad P, Buch S, Hamesch K, et al. Heterozygous carriage of the alpha1-antitrypsin Pi*Z variant increases the risk to develop liver cirrhosis. Gut 2019;68:1099-1107.
