# Supplementary material for: PNPLA3 and TM6SF2 exacerbate the impact of alcohol and metabolic dysfunction on liver fibrosis
Source: JHEP Rep. 2025 Oct 30;8(1):101649. doi: 10.1016/j.jhepr.2025.101649 (PMC12765432; doi:10.1016/j.jhepr.2025.101649)
Supplement: Multimedia component 2 [file mmc2.docx]

**JHEP Reports**

**CTAT methods**

Tables for a “Complete, Transparent, Accurate and Timely account” (CTAT) are now mandatory for all revised submissions. The aim is to enhance the reproducibility of methods.

- Only include the parts relevant to your study
- Refer to the CTAT in the main text as ‘Supplementary CTAT Table’
- Do not add subheadings
- Add as many rows as needed to include all information
- Only include one item per row

- 1. **Software**

| **Software name** | **Manufacturer** | **Version** |
| --- | --- | --- |
| R for Windows | R Core Team, R Foundation for Statistical Computing, Vienna, Austria | 4.4.1 |
|  |  |  |

- 1. **Please provide the details of the corresponding methods author for the manuscript:**

| Georg Semmler, M.D., Ph.D.  Division of Gastroenterology and Hepatology, Department of Medicine III  Medical University of Vienna,  Spitalgasse 23  1090 Vienna  Phone: +43 1 40400 47410  E-Mail: [georg.semmler@meduniwien.ac.at](mailto:georg.semmler@meduniwien.ac.at) |
| --- |
