# Supplementary material for: PNPLA3 and TM6SF2 exacerbate the impact of alcohol and metabolic dysfunction on liver fibrosis
Source: JHEP Rep. 2025 Oct 30;8(1):101649. doi: 10.1016/j.jhepr.2025.101649 (PMC12765432; doi:10.1016/j.jhepr.2025.101649)
Supplement: Multimedia component 5 [file mmc5.pdf]

Research article

JHEP|Reports

PNPLA3 and *TM6SF2* exacerbate the impact of alcohol and metabolic dysfunction on liver fibrosis

Authors

Sophie Gensluckner, Helle Lindholm Schnefeld, Jan Embacher, …, Elmar Aigner, Maja Thiele, Georg Semmler‡

Correspondence

maja.thiele@rsyd.dk (M. Thiele), georg.semmler@meduniwien.ac.at (G. Semmler).

Graphical abstract

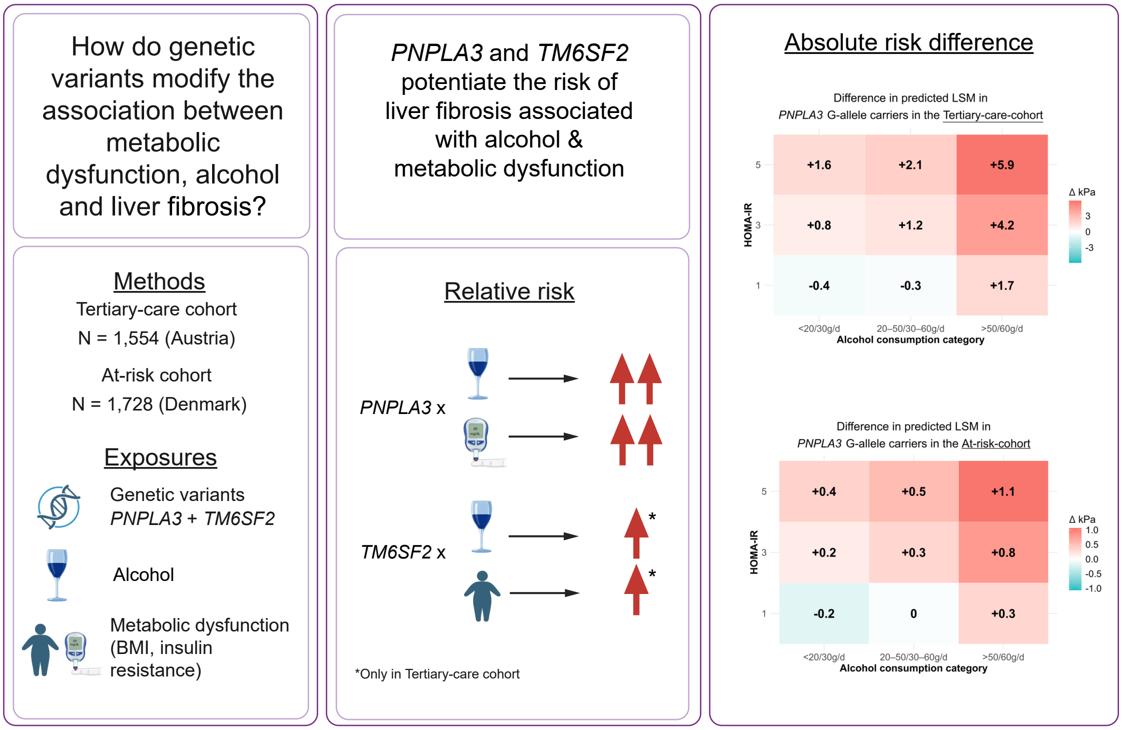

Highlights:

- Fibrosis in steatotic liver disease is mediated by environmental risk factors and genetic predisposition (*PNPLA3* and *TM6SF2).*
- Genetic variants amplify the effects of obesity, insulin resistance, and alcohol use, synergistically increasing fibrosis risk.
- These gene-environment interactions should be considered for patient counselling and risk stratification.

Impact and implications:

Our findings indicate that fibrosis progression in steatotic liver disease is mediated by an interaction of environmental risk factors (obesity, insulin resistance, alcohol consumption) and genetic risk variants, such as *PNPLA3* and *TM6SF2*, but not by genetic variants alone. This highlights the importance of acknowledging these gene–environment interactions for patient counselling and risk stratification.

https://doi.org/10.1016/j.jhepr.2025.101649
© 2025 The Author(s). Published by Elsevier B.V. on behalf of European Association for the Study of the Liver (EASL). This is an open access article under the CC BY license (http://creativecommons.org/licenses/by/4.0/). JHEP Reports, 2026, 8, 1–11

PNPLA3 and *TM6SF2* exacerbate the impact of alcohol and metabolic dysfunction on liver fibrosis☆

Sophie Gensluckner[1,†], **Helle Lindholm Schnefeld**[2,3,†], Jan Embacher[1,4,5], Camilla Dalby Hansen[2,3], Lorenz Balcar[4,5], Katrine Tholstrup Bech[2,3], Paul Thöne[4,5], Nikolaj Torp[2,3], Bernhard Wernly[1], Laura Maarit Pikkupeura[6], Stephan Zandanell[1], Christian Datz[7], Michael Strasser[1], Mads Israelsen[2,3], Mattias Mandorfer[4,5], Torben Hansen[6], Aleksander Krag[2,3], Elmar Aigner[1], Maja Thiele[2,3,*,‡], Georg Semmler[2,3,4,5,*,‡]

JHEP Reports **2026**. vol. 8 | 1–11

Check for updates

Background & Aims: Genetic predisposition (especially variants in *PNPLA3* and *TM6SF2*), metabolic dysfunction, and alcohol consumption are established risk factors for steatotic liver disease (SLD) and progression of fibrosis. However, the clinical relevance of their interaction and its implications for patient management remain unclear.

Methods: We cross-sectionally analyzed data from two cohorts: patients referred to tertiary liver care (N = 1,554) and individuals at risk for SLD (N = 1,728). Multivariable regression models with and without interaction terms were used to assess the independent and interactive effects of genetic risk variants, metabolic dysfunction (HOMA-IR, BMI), and alcohol intake on liver fibrosis severity as assessed by liver stiffness measurement (LSM).

Results: Mean age was 52 and 56 years in the Tertiary-care cohort and the At-risk cohort, respectively. Most participants were male, 23% and 53% suffered from obesity, and 39% and 58% were categorized as insulin resistant, respectively. Median LSM was 5.5 kPa and 4.7 kPa, with 21% and 9.6% having LSM ≥8 kPa, respectively. In total, 48% and 44% carried at least one *PNPLA3* G-allele (C/G or G/G), and 18% and 15% the *TM6SF2* T-allele (C/T or T/T), respectively. In multivariable regression without interaction terms, LSM was associated with HOMA-IR, alcohol consumption, BMI (At-risk cohort), *PNPLA3* and *TM6SF2*. However, when allowing for interactions, the independent effects of genetic risk variants disappeared. Instead, *PNPLA3* potentiated the association of HOMA-IR (p <0.001/p = 0.016) and severe alcohol consumption (p <0.001/p = 0.093) with LSM. *TM6SF2* amplified the effect of BMI (p = 0.006) and severe alcohol consumption (p <0.001) on LSM in the Tertiary-care-cohort.

Conclusions: Our findings indicate that *PNPLA3* and *TM6SF2* variants do not act as independent determinants of liver fibrosis once gene–environment interactions are considered. Instead, they amplify the harmful effects of metabolic dysfunction and alcohol consumption in individuals evaluated for SLD, creating a synergistic risk profile.

© 2025 The Author(s). Published by Elsevier B.V. on behalf of European Association for the Study of the Liver (EASL). This is an open access article under the CC BY license (http://creativecommons.org/licenses/by/4.0/).

Introduction

While we experience a rise of steatotic liver disease (SLD) worldwide,[1] the challenge of adequate risk stratification and counselling for patients at risk for liver-related complications remains unsolved. In addition to obesity, type 2 diabetes (T2DM), and alcohol consumption,[2,3] genetic risk variants have emerged as key contributors to disease progression,[4,5] with patatin-like phospholipase domain-containing protein 3 (*PNPLA3*) rs738409 and transmembrane 6 superfamily 2 (*TM6SF2*) rs58542926 being the best investigated variants, next to variants in hydroxysteroid 17-beta dehydrogenase 13 (*HSD17B13*), membrane-bound O-acyltransferase 7 (*MBOAT7*), glucokinase regulator protein (*GCKR*), and serpin family A member 1 (*SERPINA1*).[6] However, the incremental value of broader testing for genetic risk variants is currently unclear, calling for additional evidence to support recommendations for or against genetic testing in specific clinical scenarios where it may change patient counselling or treatment.[7]

Obesity and T2DM are both closely linked to insulin resistance and have the strongest impact on the development and progression of metabolic dysfunction-associated steatotic liver disease (MASLD).[7,8] The harmful role of alcohol consumption in the promotion of liver fibrosis is well established, with the combined effect of both alcohol and metabolic dysfunction

☆ Given their role as Associate Editors, Maja Thiele and Mattias Mandorfer had no involvement in the peer-review of this article and had no access to information regarding its peer-review. Full responsibility for the editorial process for this article was delegated to the Co-Editor Jacob George and Editor in Chief Josep M. Llovet.

* Corresponding authors. Address: FLASH Centre for Liver Research, Department of Gastroenterology and Hepatology, Odense University Hospital, J. B. Winsløws Vej 4, 5000 Odense C, Denmark; Tel.: +45 2151 3074 (M. Thiele) or Division of Gastroenterology and Hepatology, Department of Medicine III, Medical University of Vienna, Spitalgasse 23, 1090 Vienna; Austria +43 1 40400 47410. (G. Semmler)
E-mail addresses: maja.thiele@rsyd.dk (M. Thiele), georg.semmler@meduniwien.ac.at (G. Semmler).
† These authors contributed equally and share first authorship.
‡ These authors contributed equally and share last authorship.

https://doi.org/10.1016/j.jhepr.2025.101649

being many times greater than the effect of either factor alone.[9–12] In turn, the question of additional effect modification by genetic risk factors has received some attention. Specifically, studies have reported mediating effects of genetic risk variants (evidence for *PNPLA3* > polygenic risk scores > *TM6SF2* > other risk variants) and BMI, alcohol and T2DM on transaminase levels, hepatic steatosis, fibrosis/cirrhosis or liver-related outcomes in population-based[13–22] and liver-biopsy cohorts.[23,24] However, it remains unclear how these findings translate into clinical practice. While they help to understand pathophysiology, it is not clear whether these associations remain clinically relevant in settings where genotyping may actually be applied. We therefore investigated whether the presence of *PNPLA3* and *TM6SF2* modifies the impact of metabolic dysfunction and alcohol consumption on liver fibrosis in patients evaluated for SLD.

Patients and methods

Objectives

Interaction analyses focused on *PNPLA3* rs738409 and *TM6SF2* rs58542926, selected *a priori* based on the most robust evidence for fibrosis and disease progression. Other risk variants were evaluated in exploratory analyses.

Patients

Tertiary-care cohort

This cohort retrospectively included all individuals referred to the adult hepatology outpatient clinic at the University Clinic Salzburg, Austria, between 2016 and 2024, for the initial evaluation of suspected liver disease.[25,26] Individuals with other chronic liver diseases diagnosed before or after referral were excluded. Exclusion criteria were as follows: (i) missing information on alcohol consumption, (ii) missing information on insulin resistance according to homeostatic model assessment or insulin resistance (HOMA-IR) or BMI, (iii) missing data on liver stiffness measurement (LSM) by vibration-controlled transient elastography (VCTE) or invalid LSM as outlined below, (iv) missing information on genetic variants in *PNPLA3* rs738409 and *TM6SF2* rs58542926, summarized in Fig. S1.

At-risk cohort

Danish individuals aged 30-75 with risk factors for SLD (either alcohol risk group or metabolic risk group) were prospectively recruited from the general population through random personal invitations and community outreach efforts aiming at a 1:1 ratio between the two risk groups.[2] Eligibility criteria for the alcohol risk group were a self-reported alcohol consumption averaging ≥14/21 standard drinks per week over a period of at least 5 years (female/male, 12 g alcohol per standard drink). The metabolic risk group included individuals with T2DM, obesity (BMI ≥30 kg/m^2), or the presence of metabolic syndrome (as defined by the International Diabetes Federation).[27] Exclusion criteria were identical to the Tertiary-care cohort (Fig. S1).

Measurements

Tertiary-care cohort

All participants underwent a comprehensive evaluation, which included a physical examination, a detailed medical history, laboratory testing (including analysis for genetic risk variants), and assessment of fibrosis and steatosis by LSM and controlled attenuation parameter (CAP) using VCTE (FibroScan®, Echosens, France). Alcohol intake was recorded as self-reported during patient interviews.

At-risk cohort

All participants attended a screening visit conducted by experienced study staff, which included a physical examination, assessment of medical history, laboratory parameters, and VCTE. Genetic variants were obtained from stored blood samples. Alcohol intake was recorded as self-reported during patient interviews.

VCTE with LSM and CAP was performed by experienced operators adhering to established quality criteria on the day of laboratory testing.[28] Specifically, LSM was only considered reliable if <7.0 kPa or if the IQR/median was <0.3.

Definitions

The following biochemical and anthropometric definitions were applied: obesity was defined as BMI ≥30 kg/m^2, insulin resistance as HOMA-IR ≥2.5, prediabetes as HbA1c 5.7-6.4%, oral glucose tolerance test 140-199 mg/dl or fasting blood glucose 100-124 mg/dl, and diabetes was defined based on the prescription of antidiabetic drugs, HbA1c ≥6.5%, oral glucose tolerance test ≥200 mg/dl after 2 h, or fasting blood glucose ≥125 mg/dl, respectively. An LSM ≥8 kPa was considered suspected fibrosis, a CAP ≥248 dB/m denoted any steatosis and ≥280 dB/m denoted severe steatosis. Alcohol intake was categorized as <20/30 g/day, 20-50/30-60 g/day and >50/60 g/day for men/women, respectively.

Genetics

The following single nucleotide polymorphisms (SNPs) were analyzed: *PNPLA3* rs738409 C>G, *TM6SF2* rs58542926 C>T, *HSD17B13* rs72613567 T>TA (Tertiary-care cohort), *HSD17B13* rs10433937 G>A (high linkage disequilibrium r^2 = 0.96 with rs72613567;[29] At-risk cohort), *MBOAT7* rs626283 G>C (high linkage disequilibrium r^2 = 0.99 with rs641738;[30] Tertiary-care cohort), *MBOAT7* rs641738 C>T (At-risk cohort), and *SERPINA1* rs28929474 G>A.

Tertiary-care cohort

Genomic DNA was collected from peripheral blood samples according to a standardized procedure in clinical routine. The 5-nuclease allelic discrimination TaqMan genotyping method was performed using pre-designed assays from Applied Biosystems (Foster City, CA), according to the manufacturer's instructions, on a ViiA7 instrument (Applied Biosystems, Forster City, CA). For quality control, 10% of the samples were genotyped in duplicates. As genotyping was performed in clinical routine, SNPs may occasionally have failed genotyping

due to technical limitations of the assay, sequence-related complexities, or sample quality issues.

At-risk cohort

DNA was extracted from buffy coats and genotyped using the Infinium Global Screening Array v. 2.0 Beadchip (Illumina, San Diego). Genotyping was conducted on the HiScan system (Illumina) and processed with GenomeStudio software. All SNPs were imputed using the Michigan server with the 1000G Phase 3 v5 (GRCh37/hg19) reference panel. Genotyping was performed systematically at a defined timepoint for all available samples; as recruitment of this prospective cohort is ongoing, genotyping data were missing for 1,229 patients.

Ethics

Both studies were conducted in compliance with the Declaration of Helsinki, and approved by the local Ethics committees (Tertiary-care cohort: PMU-EK-2024-0059; At-risk cohort: S-20170087). All participants in the Tertiary-care cohort gave written informed consent for genetic testing, and all patients in the At-risk cohort gave written informed consent for participation in the prospective study.

Statistics

Statistical analyses were performed using R 4.4.3 (R Core Team, R Foundation for Statistical Computing, Vienna, Austria). Metric variables were expressed as mean ± standard deviation or median and IQR, as applicable, and qualitative variables as absolute numbers and relative percentages. Multivariable linear regression analyses were performed in parallel in the Tertiary-care cohort and the At-risk cohort, with LSM treated as a continuous variable and log-transformed to reduce the influence of outliers; the following independent variables were defined *a priori* as relevant confounders of LSM: age, sex, BMI, HOMA-IR, alcohol intake, *PNPLA3 G*-allele, *TM6SF2 T*-allele (*i.e.* recessive genetic models). We compared associations within a crude model without considering interaction among variables, and a model including interactions between *PNPLA3* * (BMI + HOMA-IR + alcohol intake) and

Tabel 1. Patient characteristics of the Tertiary-care cohort and At-risk cohort.

	Tertiary-care cohort (N = 1,554)	At-risk cohort (N = 1,728)
Age, years	52 ± 15	56 ± 11
Male sex	862 (56%)	867 (50%)
BMI, kg/m^2	27.0 ± 8.6	30.5 ± 6.3
BMI ≥30 kg/m^2 (obesity)	352 (23%)	920 (53%)
Diabetes	197 (13%)	315 (18%)
Prediabetes	499 (32%)	780 (45%)
HOMA-IR	2.06 [1.31-3.52]	2.90 [1.82-4.83]
HOMR-IR ≥2.5 (Insulin resistance)	612 (39%)	1,007 (58%)
Alcohol consumption		
< 20/30 g/day in ♀ /♂	1,165 (75%)	1,156 (67%)
20-50/30-60 g/day in ♀ /♂	228 (15%)	397 (23%)
>50/60 g/day in ♀ /♂	161 (10%)	175 (10%)
CAP, dB/m	276 ± 64	280 ± 57[1]
≥248 dB/m (Hepatic steatosis)	1,029 (66%)	1,188 (69%)[1]
≥280 dB/m (Severe steatosis)	776 (50%)	853 (49%)[1]
LSM, kPa	5.5 [4.3-7.2]	4.7 [3.8-5.8]
≥8 kPa (Suspected fibrosis)	328 (21%)	163 (9.4%)
PNPLA3 rs738409		
C/C	803 (52%)	971 (56%)
C/G	604 (39%)	643 (37%)
G/G	147 (9.5%)	114 (6.6%)
TM6SF2 rs58542926		
C/C	1,280 (82%)	1,474 (85%)
C/T	261 (17%)	244 (14%)
T/T	13 (0.8%)	10 (0.6%)
HSD17B13 rs72613567 (rs10433937)		
T/T (G/G)	890 (58%)[2]	869 (50%)
T/TA (G/A)	552 (36%)[2]	716 (41%)
TA/TA (A/A)	105 (6.8%)[2]	143 (8.3%)
MBOAT7 rs641738 (rs626283)		
C/C (G/G)	336 (33%)[3]	570 (33%)
C/T (G/C)	470 (46%)[3]	849 (49%)
T/T (C/C)	219 (21%)[3]	309 (18%)
SERPINA1 rs28929474		
M/M	1,454 (94%)[4]	1,656 (96%)
M/Z	98 (6.3%)[4]	72 (4.2%)

CAP, controlled attenuation parameter; HOMA-IR, homeostatic model assessment of insulin resistance; *HSD17B13*, hydroxysteroid 17-beta dehydrogenase 13; LSM, liver stiffness measurement; *MBOAT7*, membrane bound O-acyltransferase domain-containing 7; *PNPLA3*, patatin-like phospholipase domain-containing protein 3; *SERPINA1*, serpin family A member 1; *TM6SF2*, transmembrane 6 superfamily 2.
[1]Missing in 22 (1.3%).
[2]Missing in 7 (0.5%).
[3]Missing in 529 (34%).
[4]Missing in 2 (0.1%).

TM6SF2 * (BMI + HOMA-IR + alcohol intake). Results are shown as regression coefficients (β) with standard errors. Positive β-coefficients indicate higher LSM, negative values indicate lower LSM. In models with interaction terms, β-coefficients represent the direction and magnitude of modification of the main effect (*e.g.* whether the presence of a risk allele amplifies [*i.e.* synergistic effect] or attenuates [*i.e.* antagonistic effect] the effect of alcohol, BMI, or HOMA-IR). Model fit is given as adjusted R^2. For graphical display, we obtained predicted LSM from the respective model at different combinations of alcohol, BMI and HOMA-IR in carriers and non-carriers of the *PNPLA3* G-allele. The goodness-of-fit of these models was assessed using R^2, and compared using the likelihood-ratio test. As exploratory outcomes, we tested univariable associations of other available SNPs with LSM. A p value <0.05 was considered statistically significant.

Results

Patient characteristics

Patient characteristics of the Tertiary-care cohort (N = 1,554) and the At-risk cohort (N = 1,728) are summarized in Table 1.

In the Tertiary-care cohort, mean age was 52 years, 55% were male, 23% had obesity, 39% had insulin resistance, 66% had hepatic steatosis (CAP ≥248 dB/m), and 21% had suspected fibrosis (LSM ≥8 kPa). Alcohol consumption was reported as low (<20/30 g/day in women/men) in 75%, moderate (20-50/30-60 g/day in women/men) in 15% and high (>50/60 g/day in women/men) in 10%, respectively. The distribution of *PNPLA3* rs738409 genotypes was as follows: 52% wild-type (C/C), 39% heterozygous (C/G), and 9.5% homozygous for the risk allele (G/G). For *TM6SF2* rs58542926, 82% were wild-type (C/C), 17% heterozygous (C/T), and 0.8% homozygous for the risk allele (T/T). Corresponding minor allele frequencies (MAFs) were 0.29 for the *PNPLA3* G-allele, with modest deviation from the Hardy-Weinberg equilibrium (χ^2=4.55, p = 0.033), and 0.09 for the *TM6SF2* T-allele, which was in Hardy-Weinberg equilibrium (χ^2 = 0.01, p = 0.939; Table S1). The MAFs were 0.25 for *HSD17B13* TA (χ^2 = 2.34, p = 0.126), 0.44 for *MBOAT7* C (χ^2 = 5.14, p = 0.023), and 0.03 for *SERPINA1* Z (χ^2 = 1.65, p = 0.199).

In the At-risk cohort, mean age was 56 years, 50% were male, 53% had obesity, 58% had insulin resistance, 69% had hepatic steatosis, and 9.4% had suspected fibrosis. Alcohol consumption was reported as low (<20/30 g/day in women/

Table 2. Multivariable linear regression.

	Tertiary-care cohort (N = 1,554)			At-risk cohort (N = 1,728)		
**Outcome: LSM (log-transformed)**	**Reg. coeff. (β)**	**Standard error**	***p* value**	**Reg. coeff. (β)**	**Standard error**	***p* value**
**Multivariable regression without interaction term**						
Age, per 10 years	0.068	0.08	**3.25×10^{-16}**	0.017	0.009	0.059
Female sex	−0.041	0.26	0.111	−0.128	0.018	**5.09×10^{-12}**
BMI, per kg/m^2	0.001	0.001	0.456	0.011	0.002	**7.53×10^{-10}**
HOMA-IR, per log	0.238	0.016	**$<2 \times 10^{-16}$**	0.149	0.014	**$<2 \times 10^{-16}$**
Alcohol, <20/30 g/day	Ref	Ref		Ref	Ref	
Alcohol, 20-50/30-60 g/day	0.146	0.036	**4.72×10^{-5}**	0.001	0.023	0.980
Alcohol, >50/60 g/day	0.534	0.042	**$<2 \times 10^{-16}$**	0.120	0.031	**0.0001**
TM6SF2 T-allele	0.085	0.032	**0.008**	0.043	0.025	0.088
PNPLA3 G-allele	0.113	0.025	**5.01×10^{-6}**	0.050	0.018	**0.006**
		**Adjusted R^2: 0.282**			**Adjusted R^2: 0.191**	
**Multivariable regression with interaction term**						
Age, per 10 years	0.067	0.008	**4.01×10^{-16}**	0.017	0.009	**0.049**
Female sex	−0.036	0.025	0.153	−0.128	0.018	**4.39×10^{-12}**
BMI, per kg/m^2	0.003	0.002	0.271	0.011	0.002	**3.17×10^{-6}**
HOMA-IR, per log	0.143	0.023	**9.18×10^{-10}**	0.107	0.019	**2.12×10^{-8}**
Alcohol, <20/30 g/day	Ref	Ref		Ref	Ref	
Alcohol, 20-50/30-60 g/day	0.134	0.052	**0.010**	−0.021	0.032	0.501
Alcohol, >50/60 g/day	0.307	0.060	**3.72×10^{-7}**	0.076	0.044	0.088
TM6SF2 T-allele	−0.415	0.170	**0.015**	−0.165	0.144	0.252
TM6SF2 × BMI	0.018	0.007	**0.006**	0.004	0.005	0.398
TM6SF2 × log(HOMA-IR)	−0.045	0.042	0.287	0.060	0.036	0.098
TM6SF2 × Alcohol, 20-50/30-60 g/day	0.001	0.095	0.993	0.051	0.065	0.430
TM6SF2 × Alcohol, >50/60 g/day	0.418	0.103	**4.91×10^{-5}**	−0.022	0.094	0.812
PNPLA3 G-allele	0.023	0.084	0.784	−0.001	0.102	0.996
PNPLA3 × BMI	−0.004	0.003	0.218	−0.001	0.004	0.716
PNPLA3 × log(HOMA-IR)	0.189	0.031	**1.49×10^{-9}**	0.064	0.027	**0.016**
PNPLA3 × Alcohol, 20-50/30-60 g/day	0.033	0.069	0.631	0.032	0.045	0.479
PNPLA3 × Alcohol, >50/60 g/day	0.302	0.080	**0.000169**	0.104	0.062	0.093
		**Adjusted R^2: 0.313 (p = 7.69×10^{-14})**			**Adjusted R^2: 0.195 (p = 0.031)**	

Multivariable linear regression investigating factors associated with liver fibrosis as assessed by LSM (as continuous parameter, log-transformed) with and without considering an interaction (effect modification) between genetic risk variants in *PNPLA3/TM6SF2* and alcohol (semi-quantitatively), insulin resistance (as assessed by HOMA-IR as a continuous parameter, log-transformed) and obesity (as assessed by BMI as a continuous parameter). Results are shown as regression coefficients (β) with standard errors. Positive β-coefficients indicate higher LSM, negative values indicate lower LSM. In models with interaction terms, β-coefficients represent the direction and magnitude of the modification of the main effect (*e.g.* whether the presence of a risk allele amplifies or attenuates the effect of alcohol, BMI, or HOMA-IR). Model fit is given as adjusted R^2.
HOMA-IR, homeostatic model assessment of insulin resistance; LSM, liver stiffness measurement, *PNPLA3*, patatin-like phospholipase domain-containing protein 3; *TM6SF2*, transmembrane 6 superfamily 2.

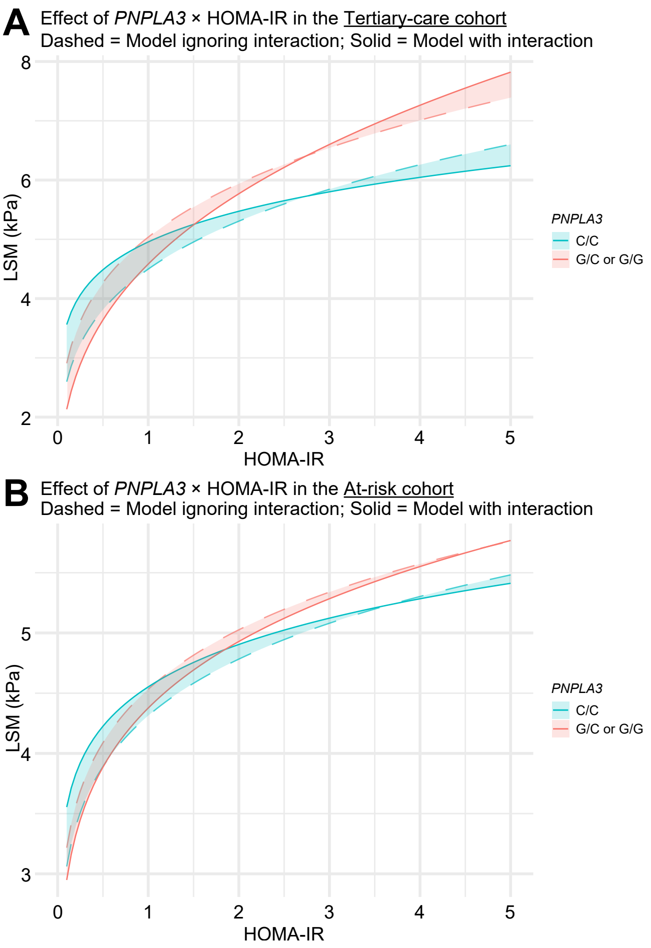

Fig. 1. Interaction plots on the association between insulin resistance (as assessed by HOMA-IR) and LSM. Interaction plots on the association between insulin resistance (as assessed by HOMA-IR) and LSM in the Tertiary-care cohort (A) and the At-risk cohort (B). The dashed line shows predictions based on the model including only the main effects (no interactions) while the solid line shows predictions including the interaction between HOMA-IR and *PNPLA3* genotype. All predictions are adjusted by fixing covariates as follows: sex (male), age (mean), BMI (mean), alcohol use (none), and *TM6SF2* wild-type (C/C). The difference between dashed and solid lines represents the additive effect (*i.e.* effect modification) captured when explicitly modeling the interaction between HOMA-IR and the *PNPLA3* G-allele. This visualizes how the *PNPLA3* G-allele modifies the association between HOMA-IR and LSM beyond what is explained by the individual (main) effect. HOMA-IR, homeostatic model assessment or insulin resistance; LSM, liver stiffness measurement; *PNPLA3*, patatin-like phospholipase domain-containing protein 3; *TM6SF2*, transmembrane 6 superfamily 2.

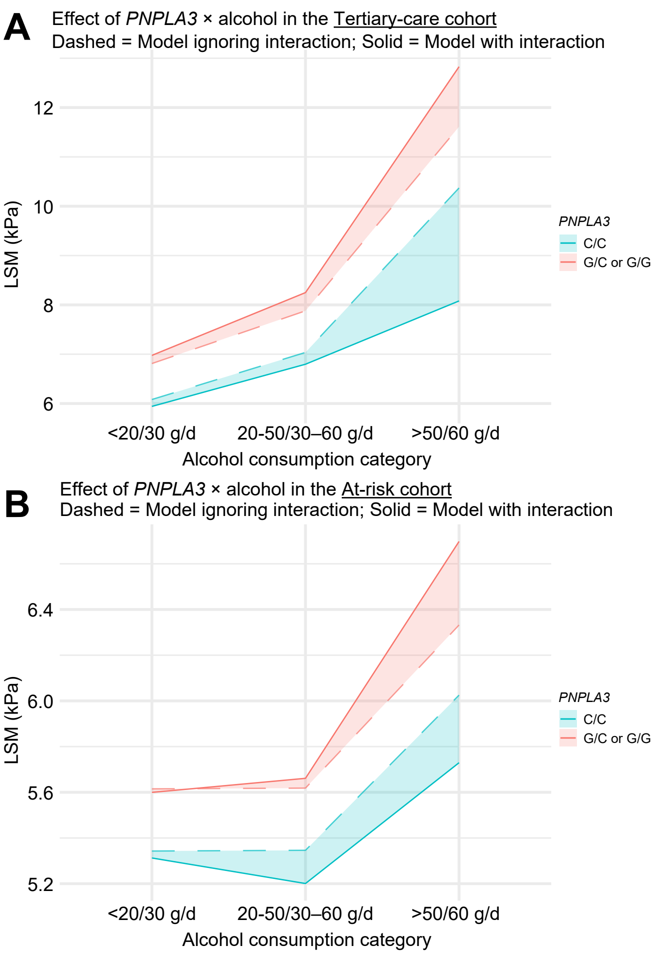

Fig. 2. Interaction plots on the association between alcohol consumption and LSM. Interaction plots on the association between alcohol consumption and LSM in the Tertiary-care cohort (A) and the At-risk cohort (B). The open dots show predictions based on the model including only the main effects (no interactions) while the filled dots show predictions including the interaction between alcohol consumption and *PNPLA3* genotype. All predictions are adjusted by fixing covariates as follows: sex (male), age (mean), BMI (mean), alcohol use (none), and *TM6SF2* wild-type (C/C). The difference between open and filled dots represents the additive effect (*i.e.* effect modification) captured when explicitly modeling the interaction between alcohol consumption and the *PNPLA3* G-allele. This visualizes how the *PNPLA3* G-allele modifies the association between alcohol use and how the *PNPLA3* G-allele modifies the association between alcohol use and LSM beyond what is explained by the individual (main) effect. LSM, liver stiffness measurement; *PNPLA3*, patatin-like phospholipase domain-containing protein 3; *TM6SF2*, transmembrane 6 superfamily 2.

men) in 67%, moderate (20–50/30–60 g/day in women/men) in 23%, and high (>50/60 g/day in women/men) in 10%, respectively. The distribution of *PNPLA3* rs738409 genotypes was as follows: 56% wild-type (C/C), 37% heterozygous (C/G), and 6.6% homozygous for the risk allele (G/G). For *TM6SF2* rs58542926, 85% were wild-type (C/C), 14% heterozygous (C/T), and 0.6% homozygous for the risk allele (T/T). The MAFs were 0.25 for *PNPLA3* G (χ^2 = 0.29, p = 0.588), 0.08 for *TM6SF2* T (χ^2 <0.001, p = 0.977), 0.29 for *HSD17B13* A (χ^2 = 0.07, p = 0.792), 0.42 for *MBOAT7* T (χ^2 = 0.05, p = 0.817), and 0.02 for *SERPINA1* Z (χ^2 = 0.78, p = 0.377).

Fibrosis risk without considering gene–environment interactions

Upon univariable analyses, risk variants in *PNPLA3* and *TM6SF2* were associated with liver fibrosis as assessed by LSM (log-transformed), with a stronger association in the Tertiary-care cohort than the At-risk cohort (Table S2). In contrast, risk variants in *HSD17B13 and MBOAT7* did not show an association, while *SERPINA1* showed an inverse association of borderline significance in the Tertiary-care cohort.

Consecutively, we studied prespecified factors known to be associated with liver fibrosis next to *PNPLA3* and *TM6SF2* risk alleles (Table 2). In the Tertiary-care cohort, age, HOMA-

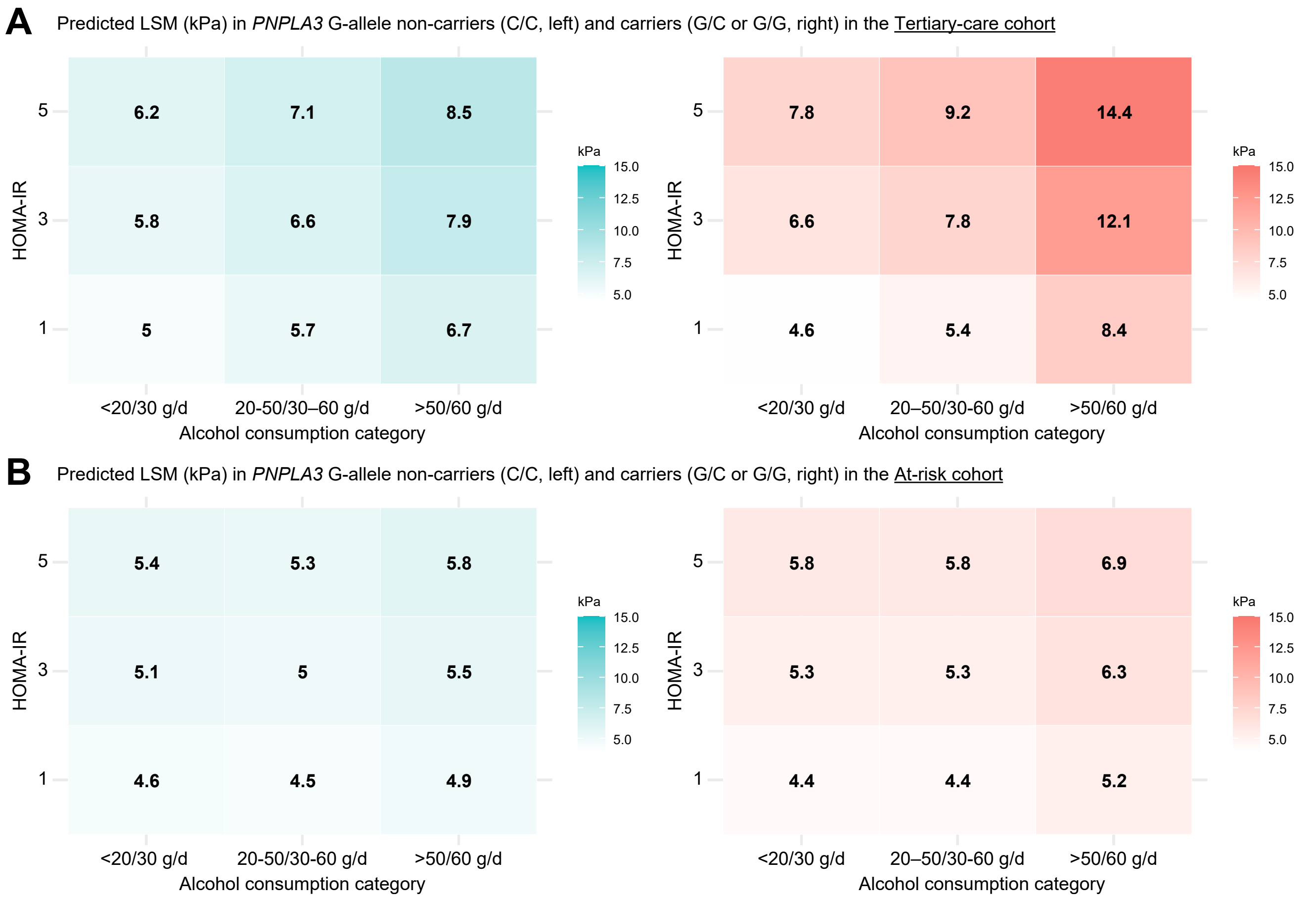

Fig. 3. Predicted LSM in *PNPLA3* G-allele carriers *vs.* non-carriers. Predicted LSM in *PNPLA3* G-allele carriers *vs.* non-carriers among combinations of alcohol consumption (<20/30 g/day, 20-50/30-60 g/day and >50/60 g/day) and HOMA-IR thresholds (1, 3 and 5 used for displaying purposes) in the Tertiary-care cohort (A) and the At-risk cohort (B). Predictions were obtained from the multivariable model, and other covariates were fixed at the reference group or mean, as applicable. HOMA-IR, homeostatic model assessment or insulin resistance; LSM, liver stiffness measurement, *PNPLA3*, patatin-like phospholipase domain-containing protein 3.

IR, moderate and severe alcohol consumption, and both the *PNPLA3* and *TM6SF2* risk alleles were independently associated with LSM in a multivariable model. In the At-risk cohort, sex, BMI, HOMA-IR, severe alcohol consumption and the *PNPLA3* G-allele were independently associated with LSM.

Fibrosis risk considering gene–environment interactions

Next, we allowed interactions between alcohol consumption and metabolic risk factors (BMI, HOMA-IR) and genetic risk variants in *PNPLA3* and *TM6SF2* to investigate the effect modification by the presence of these variants. As shown in Table 2, considering interaction terms significantly improved the overall explanatory ability of the respective models as assessed by R^2. Specifically, a stronger association between BMI and LSM was observed in carriers of the *TM6SF2* risk variant (β = 0.018, p = 0.006) in the Tertiary-care cohort, together with a more pronounced association between severe alcohol consumption and LSM (β = 0.418, p <0.001). In parallel, carrying the *PNPLA3* G-allele potentiated the association between HOMA-IR and LSM (β = 0.189, p <0.001), and severe alcohol consumption and LSM (β = 0.302, p <0.001). While alcohol consumption and HOMA-IR remained independently associated with LSM in the Tertiary-care cohort, BMI did not.

The relationship between HOMA-IR/alcohol and LSM in *PNPLA3* G-allele carriers and non-carriers is portrayed in Figs 1A and 2A, as the predicted LSM across *PNPLA3* genotypes and HOMA-IR/alcohol groups. The predicted LSMs from the full model, with and without the *PNPLA3* G-allele, are summarized in Fig. 3A: at a HOMA-IR of 1 and alcohol consumption <20/30 g/day, the predicted LSM was 4.6 kPa in those without and 5.0 kPa in those carrying the *PNPLA3* G-allele, while this difference was markedly increased at a HOMA-IR of 5 and alcohol consumption >50/60 g/day, being 8.5 kPa in those without *vs*. 14.4 kPa in those carrying the *PNPLA3* G-allele.

In the At-risk cohort, the synergistic interaction of the *PNPLA3* G-allele with HOMA-IR (β = 0.064, p = 0.016) and severe alcohol consumption (β = 0.104, p = 0.093) on LSM was confirmed, although the effect size was smaller and the improvement in the model's overall accuracy was limited. Again, a comparison of the predicted LSM in carriers *vs.* non-carriers of the *PNPLA3* G-allele is shown in Figs 1B, 2B and 3B, respectively. Importantly, the *TM6SF2* T-allele did not

change the relationship of BMI, HOMA-IR and alcohol consumption with LSM in the At-risk cohort.

In summary, neither *PNPLA3* nor *TM6SF2* remained independent risk factors *per se*, but were dependent on the presence of alcohol consumption, higher BMI or insulin resistance.

Sensitivity analyses using risk categories

To further validate interaction effects, we performed additional analyses using dichotomized clinical cut-offs for metabolic dysfunction (*i.e.* alcohol categories, BMI ≥30 kg/m^2, HOMA-IR ≥2.5; Table S3). In summary, these dichotomized interaction terms showed similar association patterns. In the Tertiary-care cohort, a more pronounced association was observed between high alcohol consumption (>50/60 g/day) and LSM (β = 0.447, p <0.001) in carriers of the *TM6SF2* T-allele, and between both high alcohol consumption (β = 0.053, p <0.001) and HOMA-IR ≥2.5 (β = 0.053, p <0.001) and LSM in carriers of the *PNPLA3* G-allele. In the At-risk cohort, there was a similarly amplified association between HOMA-IR ≥2.5 and LSM (β = 0.082, p = 0.05) in the presence of the *PNPLA3* G-allele, while the interaction for *PNPLA3* with high alcohol consumption did not attain statistical significance (β = 0.123, p = 0.055).

Discussion

Our study demonstrates that the impact of genetic risk variants in *PNPLA3* and *TM6SF2* on liver fibrosis is essentially dependent on the presence of metabolic dysfunction or significant alcohol consumption. As such, genetic risk, metabolic risk and alcohol consumption show a synergistic relationship that amplifies their individual contributions to liver fibrosis. In contrast, genetic variants *per se* did not carry an independent risk when considering these gene–environment interactions.

Genetic risk factors, metabolic dysfunction (especially central obesity and insulin resistance) and alcohol have been identified as key risk factors for the progression of SLD.[4,8,31] Recent studies have demonstrated that alcohol and metabolic dysfunction interact in a supra-additive manner, with their combined impact on fibrosis risk exceeding the sum of their individual effects.[9,10,12] Although genetic variants are often discussed as independent determinants of fibrosis,[4] their true independence is controversial. Most risk alleles converge on hepatic lipid metabolism, providing a strong biological rationale for their close relationship with metabolic dysfunction, but also for an attenuated impact on disease severity when metabolic risk factors are absent (*e.g.* when a healthy lifestyle is enforced).[6] This modifying effect is supported by the present study and the literature, summarized in Table 3 and discussed below.

At the same time, liver fibrosis progression in individuals with SLD remains highly heterogeneous, with patients of similar metabolic profiles developing fibrosis at different rates and severity.[32] From a broader perspective, some individuals may develop liver disease, while others are predominantly affected by cardiometabolic complications.[33] While factors explaining this heterogeneity are intensively investigated, gene–environment interactions will clearly be one essential piece of the puzzle that must be acknowledged.

Several previous studies have investigated interactions between genetic variants and clinical/environmental factors (summarized in Table 3). However, most of them (10/12) focused on population-based cohorts, in which the following considerations need to be acknowledged: First, implementation of genetic testing in the general population is unlikely in the near future, as it is neither cost-effective nor addresses a target population at risk for liver disease. Second, the timepoint of assessment is unstandardized, and it is unclear how these associations translate into settings where genotyping may actually be considered. Third, it is unclear whether an effect that may be visible (only) on a population-based level might also be relevant on the level of an individual patient or healthcare-provider (*e.g.* tertiary care hospital). On the other side of the spectrum, liver-biopsy cohorts (*i.e.* advanced patients already subjected to hepatology care) may in turn provide different risk estimates that cannot be extrapolated to other settings where genotyping may be considered.

In this context, our study is unique in several ways. First, it examines the interaction between *PNPLA3*/*TM6SF2* variants and metabolic dysfunction/alcohol consumption at the timepoint of evaluation for SLD and fibrosis (*i.e.* at a relevant timepoint where genetic testing may be of clinical utility and change patient counselling in terms of risk stratification, surveillance, and intensified medical treatment).[7] Here, our study provides data on the magnitude of effect, forming a basis for future cost-effectiveness analyses. Second, we used LSM as the best-established surrogate of liver fibrosis that is used for clinical decision making.[7] While limitations and a degree of uncertainty regarding fibrosis stages need to be acknowledged, LSM represents an endpoint that not only directly translates into the risk of complications of an individual patient, but that can also be monitored and changed by medical interventions.[34–37] As such, it is a clinically relevant endpoint that still allows for preventive measures to be taken (*vs.* when analyzing mortality as an endpoint), where understanding the contribution of gene–environment interactions may impact patient management. Third, we adjusted for key determinants of fibrosis (obesity and alcohol, but especially insulin resistance), thereby minimizing the risk of unmeasured confounding.

Although the current study can only report associations and can neither demonstrate causality nor the utility/cost-effectiveness of genotyping in clinical practice, it should encourage future studies that investigate more granular approaches to genetic testing. As such, genetic testing may only provide clinically meaningful additional information in patients with pronounced metabolic dysfunction (HOMA-IR, BMI) or significant alcohol consumption, as the presence of risk variants in these subgroups augments liver fibrosis. In contrast, genetic risk variants seem to marginally contribute to fibrosis risk in metabolically healthy individuals, and genetic testing is very unlikely to change the management of these patients. Sensitivity analysis on the presence or absence of obesity or insulin resistance using established cut-offs confirmed the amplifying effect in individuals within these risk categories.

Finally, we report on the MAF of genetic risk variants in settings where genotyping may be applied (see Table S1). Here, the distribution of risk alleles in At-risk cohorts (*e.g.* patients with metabolic syndrome, obesity or T2DM) seems to be comparable to the general population, while these variants reasonably accumulate in tertiary care/MASLD cohorts, especially in those undergoing liver biopsy. Interestingly, this phenomenon was most prominent for *PNPLA3* rs738409,

Table 3. Overview of studies investigating interactions between genetic risk variants and clinical/environmental factors.

Study	Design	Setting	Population	Genetic risk variants	Outcomes	Main findings	Comments
[13] Stender *et al.* (2017)	Cross-sectional	Population-based	Dallas Heart Study (DHS), Dallas Biobank, Copenhagen City Heart Study, Copenhagen General Population Study	*PNPLA3, TM6SF2, GCKR*	Hepatic steatosis (IHLC by MRS), ALT, cirrhosis (ICD 8/10; Copenhagen only)	Positive interaction between BMI and *PNPLA3/TM6SF2/GCKR* on IHLC<br>Positive interaction between BMI and *PNPLA3* on ALT	—
[14] Barata *et al.* (2019)	Cross-sectional	Population-based	14,751 individuals from 10 population-based cohorts participating in the Genetics of Obesity-Related Liver Disease (GOLD) Consortium	*PNPLA3, TM6SF2, GCKR, LYPLAL1*	Hepatic steatosis (as assessed by computed tomography)	Positive interaction between HOMA-IR/glucose/insulin/BMI/triglycerides and *PNPLA3*<br>Positive interaction between insulin/HOMA-IR/triglycerides and *GCKR*	Insulin may mediate the interaction effect of BMI, triglycerides, and glucose in individuals without diabetes
[15] Gellert-Kristensen *et al.* (2020)	Cross-sectional	Population-based	Copenhagen General Population Study, Copenhagen City Heart Study, UK Biobank	PRS combining *PNPLA3*, *TM6SF2* and *HSD17B13*	Liver biochemistry (ALT), cirrhosis and HCC (ICD 8/10)	Positive interaction between BMI/alcohol intake/diabetes and PRS on ALT<br>Positive interaction between BMI/diabetes and cirrhosis (UK biobank only)	—
[16] Gao *et al.* (2021)	Cross-sectional	Population-based, healthcare registry	UK Biobank, DiscovEHR cohort	Variants in GWAS (n = 951), PRS	ALT and AST, composite endpoint fatty liver/NAFLD/fibrosis/cirrhosis	Positive interaction between BMI and *PNPLA3/MARC1/INSR/MAU2* on AST, negative for *GCKR*, *HSD17B13* and 6 others<br>Positive interaction between BMI and *PNPLA3/TM6SF2/MARC1/SDCBP* on composite endpoint, negative for *GCKR/HSD17B13* and 7 others	Greatest interaction effect for *PNPLA3*
[17] Emdin *et al.* (2021)	Cross-sectional	Population-based, healthcare registry	UK Biobank, Partners HealthCare Biobank	PRS including *PNPLA3, TM6SF2, HSD17B13, SERPINA1, MARC1 and 7 new variants*	Cirrhosis (ICD 10)	Positive interaction between alcohol intake/BMI and PRS on cirrhosis	—
[18] Kim *et al.* (2022)	Longitudinal	Population-based	UK Biobank	*PNPLA3*	Incidence cirrhosis, HCC and liver-related death (ICD10)	Supra-additive effect of *PNPLA3* on the association between alcohol/obesity and incidence cirrhosis, HCC and liver-related death	No supra-additive effect on cardiovascular mortality
[23] Chalasani *et al.* (2024)	Longitudinal	Tertiary care	2075 individuals with biopsy-confirmed MASLD from the Clinical Research Network (MASH CRN)	*PNPLA3*	Major adverse liver outcomes (MALO)	Positive interaction between histological fibrosis stage/advanced fibrosis and *PNPLA3*<br>Positive interaction between age/T2DM/female sex and *PNPLA3*	No interaction between BMI and *PNPLA3*

(*continued on next page*)

Table 3. (*continued*)

Study	Design	Setting	Population	Genetic risk variants	Outcomes	Main findings	Comments
[19] Ghouse *et al.* (2024)	Longitudinal	Population-based	UK Biobank	35 genetic variants	Cirrhosis, HCC and liver-related mortality (ICD 9/10)	Positive interaction between BMI/alcohol consumption/ T2DM and *PNPLA3* with cirrhosis, HCC and liver-related mortality	No interaction between BMI/alcohol consumption/ T2DM and *TM6SF2, HSD17B13, MBOAT7, SERPINA1* and 30 other SNPs
[24] Jarasvaraparn *et* Vilar-Gomez *et al.* (2024)	Cross-sectional	Tertiary care	NASH Clinical Research Network (CRN) cohort	*PNPLA3*	Advanced fibrosis (histology)	Positive interaction between age/BMI/T2DM and *PNPLA3* on advanced fibrosis	—
[20] Vilar-Gomez *et al.* (2025)	Longitudinal	Population-based	4,361 individuals from NHANES III	*PNPLA3*	Liver-related death (ICD 9/10)	Positive interaction between alcohol consumption/smoking status/BMI/saturated fats/ cholesterol and *PNPLA3*<br>Negative interaction between coffee consumption and healthy eating index and *PNPLA3*	No interaction with other macronutrients
[21] Zhang *et al.* (2025)	Cross-sectional	Population-based	UK Biobank	*PNPLA3, TM6SF2* and a 16-variant PRS	IHLC by MRI-PDFF, cT1	PRS and *PNPLA3*: positive interaction with alcohol consumption, dietary quality, sedentary behavior, social connection and lifestyle score<br>*TM6SF2*: positive interaction with sedentary behavior, social connection and lifestyle score	No associations with longitudinal outcomes
[22] Xue *et al.* (2025)	Longitudinal	Population-based	UK Biobank and China Kadoorie Biobank	PRS combining *PNPLA3, TM6SF2, MBOAT7, GCKR* and *HSD17B13*	Incidence of liver-related events (LRE), HCC	Positive interaction between PRS and alcohol consumption on LRE	Interaction both in wine and non-wine consumers

ALT, alanine aminotransferase; AST, aspartate aminotransferase; GCKR, glucokinase (hexokinase 4) regulator; GWAS, genome-wide association study; HCC, hepatocellular carcinoma; HOMA-IR, homeostatic model assessment of insulin resistance; HSD17B13, hydroxysteroid 17-beta dehydrogenase 13; LRE, liver-related event; MALO, major adverse liver outcome; MBOAT7, membrane bound O-acyltransferase domain-containing 7; NAFLD, non-alcoholic fatty liver disease; PNPLA3, patatin-like phospholipase domain-containing protein 3; PRS, polygenic risk score; SERPINA1, serpin family A member 1; SLD, steatotic liver disease; TM6SF2, transmembrane 6 superfamily 2.

followed by *HSD17B13* rs72613567 and *TM6SF2* rs58542926, supporting their link with a certain phenotype (*e.g*. progressive fibrosis or elevated transaminases that lead to hepatology referral).

Importantly, this study has several limitations. First, our study can only report cross-sectional associations and cannot infer on causality, nor can it quantify utility in clinical practice. Second, although both cohorts consist of consecutive patients being evaluated in the respective settings, information on genetic variants was not available in all patients. However, this is not expected to introduce selection bias (see methods section). Third, alcohol consumption was self-reported, potentially leading to underestimation of actual alcohol intake. Fourth, confounding factors can influence LSM, and may therefore introduce noise. We intentionally studied LSM as a continuous parameter to both reduce the loss of information associated with applying binary cut-offs and to increase the granularity of the fibrosis outcome. However, it is unclear whether LSM holds the same prognostic information across its full range of measurements, especially at the lower spectrum. Fifth, the low prevalence of certain genetic risk variants increases the risk of type-2 error on a cohort-level.

In conclusion, our findings demonstrate that genetic variants in *PNPLA3* and *TM6SF2* do not act as independent risk factors for liver fibrosis, but amplify the detrimental effects of metabolic dysfunction (especially insulin resistance) and alcohol consumption. These gene–environment interactions demonstrate a synergistic effect in promoting liver fibrosis and underscore the importance of integrating this knowledge into patient counselling and risk stratification.

Affiliations

[1]*First Department of Medicine, University Clinic Salzburg, Paracelsus Medical University Salzburg, Salzburg, Austria;* [2]*Centre for Liver Research, Department of Gastroenterology and Hepatology, Odense University Hospital, Odense, Denmark;* [3]*Institute of Clinical Research, Faculty of Health Sciences, University of Southern Denmark, Odense, Denmark;* [4]*Division of Gastroenterology and Hepatology, Department of Medicine III, Medical University of Vienna, Vienna, Austria;* [5]*Vienna Hepatic Hemodynamic Lab, Division of Gastroenterology and Hepatology, Department of Medicine III, Medical University of Vienna, Vienna, Austria;* [6]*Novo Nordisk Foundation Center for Basic Metabolic Research, Faculty of Health and Medical Sciences, University of Copenhagen, Copenhagen, Denmark;* [7]*Department of Internal Medicine, General Hospital Oberndorf, Teaching Hospital of the Paracelsus Medical University Salzburg, Oberndorf, Salzburg, Austria*

Abbreviations

CAP, controlled attenuation parameter; *GCKR*, glucokinase (hexokinase 4) regulator; HOMA-IR, homeostatic model assessment or insulin resistance; *HSD17B13,* hydroxysteroid 17-beta dehydrogenase 13; LSM, liver stiffness measurement; MAF, minor allele frequency; MASLD, metabolic dysfunction-associated steatotic liver disease; *MBOAT7*, membrane bound O-acyltransferase domain-containing 7; *PNPLA3*, patatin-like phospholipase domain-containing protein 3; *SERPINA1*, serpin family A member 1; SLD, steatotic liver disease; SNPs, single nucleotide polymorphisms; T2DM, type 2 diabetes mellitus;*TM6SF2*, transmembrane 6 superfamily 2; VCTE, vibration-controlled transient elastography.

Financial support

This study was supported by a grant from the Novo Nordisk Foundation (NNF20OC0059393). The funder had no role in the decision to write or submit this work for publication. J.E., P.T., L.B., M.M. and G.S. are supported by the Clinical Research Group MOTION, Medical University of Vienna, Vienna, Austria – a project funded by the Clinical Research Groups Program of the Ludwig Boltzmann Gesellschaft (Grant Nr: LBG_KFG_22_32) with funds from the Fonds Zukunft Österreich.

Conflict of interest

S.G. received travel support from Ipsen, Roche, Galapagos, Gilead. M.M. received grant support from Echosens, served as a consultant and/or advisory board member and/or speaker for AbbVie, Collective Acumen, Echosens, Gilead, Ipsen, Takeda, and W. L. Gore & Associates and received travel support from AbbVie and Gilead. E.A. received grant support from Intercept, Sanofi-Genzyme, Takeda, and Alexion; honoraria from Alnylam, Gilead, Intercept, Takeda, Sanofi, Mirum, Amgen, Novartis, Sobi, and Amicus. M.T. speakers fee from Echosens, Madrigal, Takeda, and Novo Nordisk. Advisory fee from Boehringer Ingelheim, Astra Zeneca, Novo Nordisk and GSK. Research grant from GSK. Co-founder and board member for Evido. Board member for Alcohol & Society (non-governmental organisation). Funded by a grant from the Novo Nordisk Foundation (NNF20OC0059393). G.S. received travel support from Amgen. All other authors have nothing to disclose.
Please refer to the accompanying ICMJE disclosure forms for further details.

Authors' contributions

Study concept and design (S.G., C.D.H., M.T., G.S.), acquisition of data (all authors), analysis and interpretation of data (S.G., C.D.H., M.T., G.S.), drafting of the manuscript (S.G., C.D.H., M.T., G.S.) critical revision of the manuscript for important intellectual content (all authors).

Declaration of AI and AI-assisted technologies in the writing process

During the preparation of this work the authors used ChatGPT (OpenAI) for language editing and rephrasing, without contributing to the scientific content or generation of ideas. After using this tool/service, the authors reviewed and edited the content as needed and take full responsibility for the content of the publication.

Data availability

Data are available from the corresponding authors upon reasonable request and adhering to European data protection laws and local regulatory restrictions.

Supplementary data

Supplementary data to this article can be found online at https://doi.org/10.1016/j.jhepr.2025.101649.

References

Author names in bold designate shared co-first authorship.

[1] Feng G, Targher G, Byrne CD, et al. Global burden of metabolic dysfunction-associated steatotic liver disease, 2010 to 2021. JHEP Rep 2025;7:101271.
[2] Hansen CD, Hansen JK, Israelsen M, et al. Prevalence, severity and determinants of steatotic liver disease among individuals with metabolic and alcohol risk from the community. J Hepatol 2025;83(6):1278–1291.
[3] **Semmler G**, **Thöne P**, Embacher J, et al. Prevalence of fibrosis and applicability of lab-based non-invasive tests from primary to tertiary care. Clin Gastroenterol Hepatol 2025. Online ahead of print.
[4] Luukkonen PK, Qadri S, Ahlholm N, et al. Distinct contributions of metabolic dysfunction and genetic risk factors in the pathogenesis of non-alcoholic fatty liver disease. J Hepatol 2022;76:526–535.
[5] Israelsen M, Juel HB, Detlefsen S, et al. Metabolic and genetic risk factors are the strongest predictors of severity of alcohol-related liver fibrosis. Clin Gastroenterol Hepatol 2022;20:1784–1794.e1789.
[6] Sookoian S, Rotman Y, Valenti L. Genetics of metabolic dysfunction-associated steatotic liver disease: the state of the art update. Clin Gastroenterol Hepatol : official Clin Pract J Am Gastroenterological Assoc 2024;22:2177–2187.e2173.
[7] EASL-EASD-EASO Clinical Practice Guidelines on the management of metabolic dysfunction-associated steatotic liver disease (MASLD). J Hepatol 2024;81:492–542.

[8] **Semmler G**, **Balcar L**, Wernly S, et al. Insulin resistance and central obesity determine hepatic steatosis and explain cardiovascular risk in steatotic liver disease. Front Endocrinol 2023;14:1244405.
[9] Hart CL, Morrison DS, Batty GD, et al. Effect of body mass index and alcohol consumption on liver disease: analysis of data from two prospective cohort studies. BMJ (Clinical research ed) 2010;340:c1240.
[10] **Marti-Aguado D**, **Calleja JL**, Vilar-Gomez E, et al. Low-to-moderate alcohol consumption is associated with increased fibrosis in individuals with metabolic dysfunction-associated steatotic liver disease. J Hepatol 2024;81:930–940.
[11] Ochoa-Allemant P, Hubbard RA, Kaplan DE, et al. Adverse liver outcomes, cardiovascular events, and mortality in steatotic liver disease. JAMA Intern Med 2025;185:986–995.
[12] Hagström H, Hegmar H, Moreno C. Interactions between the metabolic syndrome and alcohol consumption increases the risk of liver disease. United Eur Gastroenterol J 2024;12:168–176.
[13] Stender S, Kozlitina J, Nordestgaard BG, et al. Adiposity amplifies the genetic risk of fatty liver disease conferred by multiple loci. Nat Genet 2017;49:842–847.
[14] Barata L, Feitosa MF, Bielak LF, et al. Insulin resistance exacerbates genetic predisposition to nonalcoholic fatty liver disease in individuals without diabetes. Hepatol Commun 2019;3:894–907.
[15] Gellert-Kristensen H, Richardson TG, Davey Smith G, et al. Combined effect of PNPLA3, TM6SF2, and HSD17B13 variants on risk of cirrhosis and hepatocellular carcinoma in the general population. Hepatology (Baltimore, Md) 2020;72:845–856.
[16] Gao C, Marcketta A, Backman JD, et al. Genome-wide association analysis of serum alanine and aspartate aminotransferase, and the modifying effects of BMI in 388k European individuals. Genet Epidemiol 2021;45:664–681.
[17] Emdin CA, Haas M, Ajmera V, et al. Association of genetic variation with cirrhosis: a multi-trait genome-wide association and gene-environment interaction study. Gastroenterology 2021;160:1620–1633.e1613.
[18] Kim HS, Xiao X, Byun J, et al. Synergistic associations of PNPLA3 I148M variant, alcohol intake, and obesity with risk of cirrhosis, hepatocellular carcinoma, and mortality. JAMA Netw open 2022;5:e2234221.
[19] Ghouse J, Sveinbjörnsson G, Vujkovic M, et al. Integrative common and rare variant analyses provide insights into the genetic architecture of liver cirrhosis. Nat Genet 2024;56:827–837.
[20] Vilar-Gomez E, Gawrieh S, Vuppalanchi R, et al. PNPLA3 rs738409, environmental factors and liver-related mortality in the US population. J Hepatol 2025;82:571–581.
[21] Zhang X, Wang H, Guo C, et al. Genetic risk amplifies lifestyle effects on hepatic steatosis and its progression: insights from a population-based cohort. Dig Liver Dis : official J Ital Soc Gastroenterol Ital Assoc Study Liver 2025;57:893–901.
[22] **Xue H**, **Wang L**, Sun D, et al. Associations of alcohol consumption and genetic predisposition to hepatic steatosis with liver-related events: results from large population-based cohort studies. Gastroenterology 2025;169 (4):705–714.
[23] Chalasani N, Vilar-Gomez E, Loomba R, et al. PNPLA3 rs738409, age, diabetes, sex, and advanced fibrosis jointly contribute to the risk of major adverse liver outcomes in metabolic dysfunction-associated steatotic liver disease. Hepatology (Baltimore, Md) 2024;80:1212–1226.
[24] **Jarasvaraparn C**, **Vilar-Gomez E**, Yates KP, et al. Age, BMI, and type 2 diabetes modify the relationship between PNPLA3 and advanced fibrosis in children and adults with NAFLD. Clin Gastroenterol Hepatol 2024;22:1024–1036.e1022.
[25] **Balcar L**, **Semmler G**, Oberkofler H, et al. PNPLA3 is the dominant SNP linked to liver disease severity at time of first referral to a tertiary center. Dig Liver Dis : official J Ital Soc Gastroenterol Ital Assoc Study Liver 2022;54:84–90.
[26] Semmler G, Balcar L, Simbrunner B, et al. Diagnostic and prognostic performance of the LiverRisk score in tertiary care. JHEP Rep 2024;6.
[27] Alberti KG, Eckel RH, Grundy SM, et al. Harmonizing the metabolic syndrome: a joint interim statement of the international diabetes federation task force on epidemiology and prevention; national heart, lung, and blood institute; American heart association; world heart federation; international atherosclerosis society; and international association for the study of obesity. Circulation 2009;120:1640–1645.
[28] Boursier J, Zarski JP, de Ledinghen V, et al. Determination of reliability criteria for liver stiffness evaluation by transient elastography. Hepatology 2013;57:1182–1191. Baltimore, Md.
[29] Basyte-Bacevice V, Skieceviciene J, Valantiene I, et al. SERPINA1 and HSD17B13 gene variants in patients with liver fibrosis and cirrhosis. J Gastrointest Liver Dis : JGLD 2019;28:297–302.
[30] Derived from European population of the 1000 Genomes Project (https://ldlink.nih.gov/, accessed 31.August.2025).
[31] Bril F, Kalavalapalli S, Lomonaco R, et al. Insulin resistance is an integral feature of MASLD even in the presence of PNPLA3 variants. JHEP Rep : innovation Hepatol 2024;6:101092.
[32] Arrese M, Arab JP, Barrera F, et al. Insights into nonalcoholic fatty-liver disease heterogeneity. Semin Liver Dis 2021;41:421–434.
[33] **Raverdy V**, **Tavaglione F**, **Chatelain E**, et al. Data-driven cluster analysis identifies distinct types of metabolic dysfunction-associated steatotic liver disease. Nat Med 2024;30:3624–3633.
[34] Semmler G, Yang Z, Fritz L, et al. Dynamics in liver stiffness measurements predict outcomes in advanced chronic liver disease. Gastroenterology 2023;165:1041–1052.
[35] Thorhauge KH, Semmler G, Johansen S, et al. Using liver stiffness to predict and monitor the risk of decompensation and mortality in patients with alcohol-related liver disease. J Hepatol 2024;81(1):23–32.
[36] Gawrieh S, Vilar-Gomez E, Wilson LA, et al. Increases and decreases in liver stiffness measurement are independently associated with the risk of liver-related events in NAFLD. J Hepatol 2024;81:600–608.
[37] Lin H, Lee HW, Yip TC-F, et al. Vibration-controlled transient elastography scores to predict liver-related events in steatotic liver disease. Jama 2024;331(15):1287–1297.

Keywords: *PNPLA3*; *TM6SF2*; Liver fibrosis; Steatotic liver disease; Alcohol; Metabolic dysfunction.
Received 16 June 2025; received in revised form 14 October 2025; accepted 16 October 2025; Available online 30 October 2025

Journal of Hepatology, Volume *8*

Supplemental information

PNPLA3 and *TM6SF2* exacerbate the impact of alcohol and metabolic dysfunction on liver fibrosis

Sophie Gensluckner, Helle Lindholm Schnefeld, Jan Embacher, Camilla Dalby Hansen, Lorenz Balcar, Katrine Tholstrup Bech, Paul Thöne, Nikolaj Torp, Bernhard Wernly, Laura Maarit Pikkupeura, Stephan Zandanell, Christian Datz, Michael Strasser, Mads Israelsen, Mattias Mandorfer, Torben Hansen, Aleksander Krag, Elmar Aigner, Maja Thiele, and Georg Semmler

PNPLA3 and TM6SF2 exacerbate the impact of alcohol and metabolic dysfunction on liver fibrosis

Sophie Gensluckner, Helle Lindholm Schnefeld, Jan Embacher, Camilla Dalby Hansen, Lorenz Balcar, Katrine Tholstrup Bech, Paul Thöne, Nikolaj Torp, Bernhard Wernly, Laura Maarit Pikkupeura, Stephan Zandanell, Christian Datz, Michael Strasser, Mads Israelsen, Mattias Mandorfer, Torben Hansen, Aleksander Krag, Elmar Aigner, Maja Thiele, Georg Semmler

Table of contents

Fig. S1……………………………………………………………………………......2

Table S1……………………………………………………………………………3

Table S2……………………………………………………………………………5

Table S3……………………………………………………………………………6

Supplementary references………………………………………………………....7

Fig. S1. Patient flowchart for the tertiary-care-cohort (n=1554) and at-risk-cohort (n=1728).

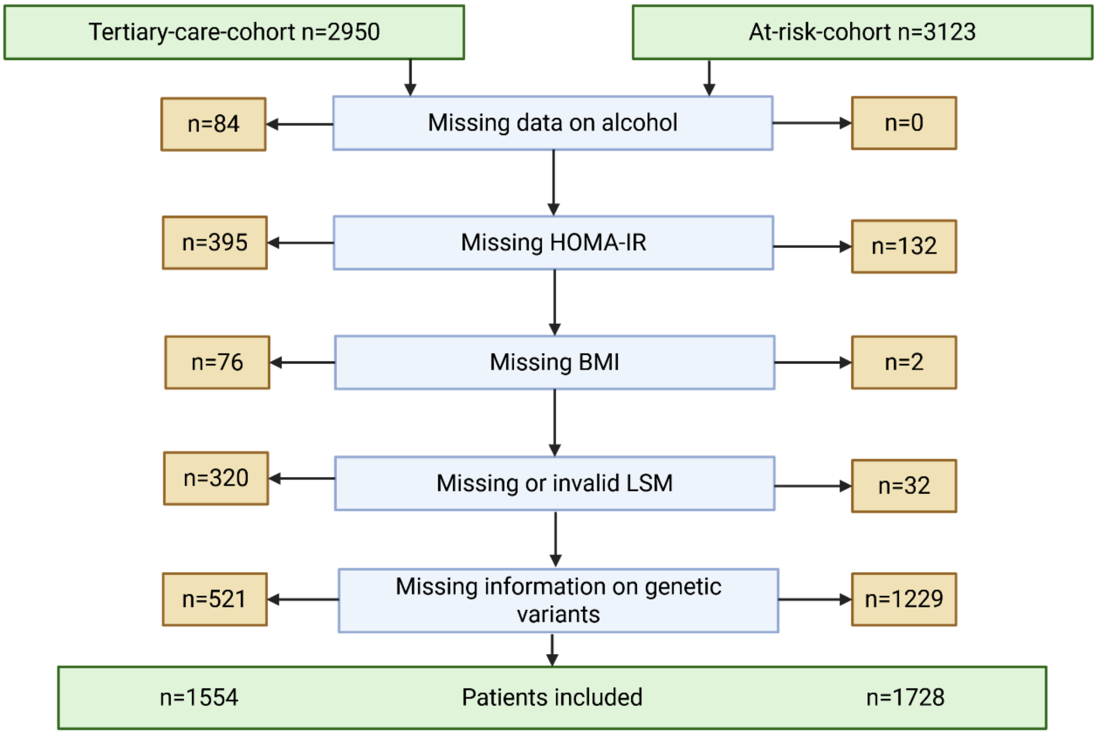

Abbreviations: HOMA-IR – Homeostatic model assessment or insulin resistance; BMI – body mass index; LSM – liver stiffness measurement

Table S1. Distribution of genetic risk variants in European populations the literature and the current study cohorts. Cohorts were sorted in respective settings according to minor allele frequency (MAF).

Gene	Setting	Study	Wildtype (%)	Heterozygous minor allele (%)	Homozygous minor allele (%)	MAF (%)
PNPLA3 rs738409	General population	[1]	66	31	2.8	0.18
		[2, 3] (UK Biobank)	62	34	4.7-4.8	0.22
		1000 Genomes Project[1]	-	-	-	0.23
		[4]	-	-	-	0.23
		[5]	59	38	3.8	0.23
		[3]	60	35	5.1	0.23
		[6]	60	36	4.6	0.23
		[7]	-	-	-	0.24
		[8]	53	39	8.4	0.28
	At-risk population	**At-risk cohort**	**56**	**37**	**6.6**	**0.25**
		[9]	57	34	9.4	0.26
	Tertiary care	**Tertiary-care-cohort**	**52**	**39**	**9.5**	**0.29**
		[1]	41-44	44-45	12-14	0.34-0.37
		[10]	44	43	14	0.36
		[11]	42	43	15	0.37
		[12] (median + range from meta-analysis)	38 (25-78)	41 (15-49)	17 (6.9-36)	0.40
TM6SF2 rs58542926	General population	[13]	-	-	-	0.07
		1000 Genomes Project[1]	-	-	-	0.07
		[7]	-	-	-	0.07
		[6]	81	14	0.7	0.08
		[8]	84	15	0.6	0.08
		[3] (UK Biobank)	85	14	0.6	0.08
		[3]	83	16	0.8	0.09
	At-risk population	**At-risk cohort**	**85**	**14**	**0.6**	**0.08**
	Tertiary care	**Tertiary-care-cohort**	**82**	**17**	**0.8**	**0.09**
		[11]	79	19	1.8	0.11
		[10]	78	20	2.3	0.12
MBOAT7	General population	[14][4]	34	49	18	0.42
		[7]	-	-	-	0.43
		1000 Genomes Project[1]	-	-	-	0.44

	At-risk population	**At-risk cohort**	**33**	**49**	**18**	**0.42**
		[15]	30	52	17	0.43
	Tertiary care	**Tertiary-care-cohort**	**33**	**46**	**21**	**0.44**
		[14]	33	46	21	0.44
		[16]	34	45	21	0.44
		[17]	29	50	21	0.46
		[11]	31	47	22	0.46
HSD17B13 rs72613567 (rs10433937)[3]	General population	[3] (UK Biobank)	48	43	9.6	0.31
		[3]	50	40	7.8	0.28
	At-risk population	**At-risk cohort**	**50**	**41**	**8.3**	**0.29**
	Tertiary care	**Tertiary-care-cohort**	**58**	**36**	**6.8**	**0.25**
		[18]	-	-	-	0.21
		[19]	-	-	-	0.19
SERPINA1 rs28929474	General population	1000 Genomes Project[1]	-	-	-	0.02
		[20] (UK Biobank)	96	4	0.04	0.02
	At-risk population	**At-risk cohort**	**96**	**4.2**	**0**	**0.02**
	Tertiary care	[21]	96	3.8	0.14	0.02
		**Tertiary-care-cohort**	**94**	**6.3**	**-[5]**	**0.03[5]**

[1] Derived from European population (n=503) from the 1000 Genomes project, assessed from https://ldlink.nih.gov/; [2] High linkage disequilibrium, r^2=0.99; [3] High linkage disequilibrium, r^2=0.99; [4] Based on data from European Americans; [5] As carrying the homozygous risk allele denotes alpha-1 antitrypsin deficiency, these individuals were a priori excluded from the study cohort. MAF was calculated assuming n=0 for Z/Z.

Abbreviations: *PNPLA3* - Patatin-like phospholipase domain-containing protein 3, *TM6SF2* - Transmembrane 6 superfamily 2, *MBOAT7* - Membrane Bound O-Acyltransferase Domain Containing 7, *HSD17B13* - Hydroxysteroid 17-Beta Dehydrogenase 13, *SERPINA1* - Serpin Family A Member 1, MAF - minor allele frequency

Table S2. Univariable linear regression analysis studying the association of genetic risk variants with liver fibrosis assessed by LSM (log-transformed).

	Tertiary-care-cohort (n=1554)			At-risk-cohort (n=1728)		
	Estimate (β)	Standard Error	p-value	Estimate (β)	Standard Error	p-value
PNPLA3 G-allele	0.145	0.029	**5.24×10^{-7}**	0.061	0.020	**0.002**
TM6SF2 T-allele	0.113	0.038	**0.003**	0.054	0.028	0.054
HSD17B13 TA-allele (A-allele)	0.020	0.029	0.495	0.004	0.020	0.848
MBOAT7 T-allele (C-allele)	0.038	0.039	0.323	0.012	0.021	0.561
SERPINA1 Z-allele	-0.120	0.061	**0.047**	0.050	0.050	0.315

Abbreviations: LSM – liver stiffness measurement, *PNPLA3* - Patatin-like phospholipase domain-containing protein 3, *TM6SF2* - Transmembrane 6 superfamily 2, *MBOAT7* - Membrane Bound O-Acyltransferase Domain Containing 7, *HSD17B13* - Hydroxysteroid 17-Beta Dehydrogenase 13, *SERPINA1* - Serpin Family A Member 1, MAF - minor allele frequency

Table S3. Multivariable linear regression investigating factors associated with liver fibrosis assessed by LSM (log-transformed) with and without considering an interaction / effect modification of genetic risk variants *PNPLA3* and *TM6SF2* and alcohol (semi-quantitatively), insulin resistance (as assessed by HOMA-IR ≥2.5) and obesity (BMI ≥30kg/m²).

	**Tertiary-care-cohort (n=1554)**			**At-risk-cohort (n=1728)**		
	**Estimate (β)**	**Standard Error**	**p-value**	**Estimate (β)**	**Standard Error**	**p-value**
**Multivariable regression with interaction term**						
Age, per 10 years	0.076	0.008	**$< 2\times10^{-16}$**	0.019	0.009	**0.033**
Female sex	-0.053	0.026	**0.039**	-0.138	0.019	**4.96×10^{-13}**
Obesity (BMI ≥30kg/m²)	0.231	0.047	**1.01×10^{-6}**	0.138	0.029	**2.98×10^{-6}**
Insulin resistance (HOMA-IR ≥2.5)	0.193	0.039	**9.33×10^{-7}**	0.100	0.029	**0.001**
Alcohol, 20-50/30-60g/d	0.125	0.053	**0.019**	-0.04	0.033	0.218
Alcohol, >50/60g/d	0.282	0.061	**4.60×10^{-6}**	0.062	0.046	0.180
TM6SF2 T-allele	0.032	0.047	0.496	-0.043	0.052	0.407
TM6SF2 × Obesity	0.117	0.077	0.126	0.07	0.058	0.228
TM6SF2 × Insulin resistance	-0.069	0.068	0.315	0.093	0.058	0.108
TM6SF2 × Alcohol, 20-50/30-60g/d	-0.033	0.097	0.738	0.050	0.067	0.457
TM6SF2 × Alcohol, >50/60g/d	0.447	0.105	**2.01×10^{-5}**	-0.037	0.098	0.708
PNPLA3 G-allele	0.02	0.036	0.576	-0.027	0.039	0.48
PNPLA3 × Obesity	-0.157	0.062	**0.011**	0.021	0.042	0.624
PNPLA3 × Insulin resistance	0.227	0.053	**1.75×10^{-5}**	0.082	0.042	**0.05**
PNPLA3 × Alcohol, 20-50/30-60g/d	0.019	0.070	0.783	0.029	0.047	0.536
PNPLA3 × Alcohol, >50/60g/d	0.316	0.081	**0.0001**	0.123	0.064	0.055
	**Adjusted R^2: 0.288**			**Adjusted R^2: 0.132**		

<u>Abbreviations</u>: LSM – liver stiffness measurement, *PNPLA3* - Patatin-like phospholipase domain-containing protein 3, *TM6SF2* - Transmembrane 6 superfamily 2, HOMA-IR - Homeostatic model assessment or insulin resistance, BMI – body mass index

Supplementary references

Author names in bold designate shared co-first authorship

[1] Valenti L, Al-Serri A, Daly AK, et al. Homozygosity for the patatin-like phospholipase-3/adiponutrin I148M polymorphism influences liver fibrosis in patients with nonalcoholic fatty liver disease†. Hepatology (Baltimore, Md) 2010;51:1209-1217.

[2] Kim H-s, Xiao X, Byun J, et al. Synergistic Associations of PNPLA3 I148M Variant, Alcohol Intake, and Obesity With Risk of Cirrhosis, Hepatocellular Carcinoma, and Mortality. JAMA network open 2022;5:e2234221-e2234221.

[3] Gellert-Kristensen H, Richardson TG, Davey Smith G, et al. Combined Effect of PNPLA3, TM6SF2, and HSD17B13 Variants on Risk of Cirrhosis and Hepatocellular Carcinoma in the General Population. Hepatology (Baltimore, Md) 2020;72:845-856.

[4] Kozlitina J, Sookoian S. Global Epidemiological Impact of PNPLA3 I148M on Liver Disease. Liver international : official journal of the International Association for the Study of the Liver 2025;45:e16123.

[5] Romeo S, Kozlitina J, Xing C, et al. Genetic variation in PNPLA3 confers susceptibility to nonalcoholic fatty liver disease. Nature genetics 2008;40:1461-1465.

[6] Köpp J, Fleßa S, Lieb W, et al. Association of PNPLA3 rs738409 and TM6SF2 rs58542926 with health services utilization in a population-based study. BMC Health Services Research 2016;16:41.

[7] Simons N, Isaacs A, Koek GH, et al. PNPLA3, TM6SF2, and MBOAT7 Genotypes and Coronary Artery Disease. Gastroenterology 2017;152:912-913.

[8] **Semmler G, Balcar L**, Wernly S, et al. No association of NAFLD-related polymorphisms in PNPLA3 and TM6SF2 with all-cause and cardiovascular mortality in an Austrian population study. Wiener klinische Wochenschrift 2024;136:251-257.

[9] Kantartzis K, Peter A, Machicao F, et al. Dissociation between fatty liver and insulin resistance in humans carrying a variant of the patatin-like phospholipase 3 gene. Diabetes 2009;58:2616-2623.

[10] Liu YL, Reeves HL, Burt AD, et al. TM6SF2 rs58542926 influences hepatic fibrosis progression in patients with non-alcoholic fatty liver disease. Nat Commun 2014;5:4309.

[11] Krawczyk M, Rau M, Schattenberg JM, et al. Combined effects of the PNPLA3 rs738409, TM6SF2 rs58542926, and MBOAT7 rs641738 variants on NAFLD severity: a multicenter biopsy-based study1. Journal of lipid research 2017;58:247-255.

[12] Zhao Y, Zhao W, Ma J, et al. Patatin-like phospholipase domain-containing 3 gene (PNPLA3) polymorphic (rs738409) single nucleotide polymorphisms and susceptibility to nonalcoholic fatty liver disease: A meta-analysis of twenty studies. Medicine 2023;102:e33110.

[13] Kozlitina J, Smagris E, Stender S, et al. Exome-wide association study identifies a TM6SF2 variant that confers susceptibility to nonalcoholic fatty liver disease. Nat Genet 2014;46:352-356.

[14] **Mancina RM, Dongiovanni P**, Petta S, et al. The MBOAT7-TMC4 Variant rs641738 Increases Risk of Nonalcoholic Fatty Liver Disease in Individuals of European Descent. Gastroenterology 2016;150:1219-1230.e1216.

[15] Luukkonen PK, Zhou Y, Hyötyläinen T, et al. The MBOAT7 variant rs641738 alters hepatic phosphatidylinositols and increases severity of non-alcoholic fatty liver disease in humans. Journal of hepatology 2016;65:1263-1265.

[16] Donati B, Dongiovanni P, Romeo S, et al. MBOAT7 rs641738 variant and hepatocellular carcinoma in non-cirrhotic individuals. Scientific Reports 2017;7:4492.

[17] Thangapandi VR, Knittelfelder O, Brosch M, et al. Loss of hepatic Mboat7 leads to liver fibrosis. Gut 2021;70:940-950.

[18] Anstee QM, Darlay R, Cockell S, et al. Genome-wide association study of non-alcoholic fatty liver and steatohepatitis in a histologically characterised cohort(☆). Journal of hepatology 2020;73:505-515.

[19] Ma Y, Belyaeva OV, Brown PM, et al. 17-Beta Hydroxysteroid Dehydrogenase 13 Is a Hepatic Retinol Dehydrogenase Associated With Histological Features of Nonalcoholic Fatty Liver Disease. Hepatology (Baltimore, Md) 2019;69:1504-1519.

[20] Hakim A, Moll M, Qiao D, et al. Heterozygosity of the Alpha 1-Antitrypsin Pi*Z Allele and Risk of Liver Disease. Hepatology communications 2021;5:1348-1361.

[21] Strnad P, Buch S, Hamesch K, et al. Heterozygous carriage of the alpha1-antitrypsin Pi*Z variant increases the risk to develop liver cirrhosis. Gut 2019;68:1099-1107.
